# Public comment sentiment on educational videos: Understanding the effects of presenter gender, video format, threading, and moderation on YouTube TED talk comments

**DOI:** 10.1371/journal.pone.0197331

**Published:** 2018-06-01

**Authors:** George Veletsianos, Royce Kimmons, Ross Larsen, Tonia A. Dousay, Patrick R. Lowenthal

**Affiliations:** 1 School of Education & Technology, Royal Roads University, Victoria, BC, Canada; 2 Department of Instructional Psychology and Technology, Brigham Young University, Provo, UT, United States of America; 3 Department of Curriculum and Instruction, University of Idaho, Moscow, ID, United States of America; 4 Department of Educational Technology, Boise State University, Boise, ID, United States of America; Indiana University Bloomington, UNITED STATES

## Abstract

Scholars, educators, and students are increasingly encouraged to participate in online spaces. While the current literature highlights the potential positive outcomes of such participation, little research exists on the sentiment that these individuals may face online and on the factors that may lead some people to face different types of sentiment than others. To investigate these issues, we examined the strength of positive and negative sentiment expressed in response to TEDx and TED-Ed talks posted on YouTube (*n* = 655), the effect of several variables on comment and reply sentiment (*n* = 774,939), and the projected effects that sentiment-based moderation would have had on posted content. We found that most comments and replies were neutral in nature and some topics were more likely than others to elicit positive or negative sentiment. Videos of male presenters showed greater neutrality, while videos of female presenters saw significantly greater positive and negative polarity in replies. Animations neutralized both the negativity and positivity of replies at a very high rate. Gender and video format influenced the sentiment of replies and not just the initial comments that were directed toward the video. Finally, we found that using sentiment as a way to moderate offensive content would have a significant effect on non-offensive content. These findings have far-reaching implications for social media platforms and for those who encourage or prepare students and scholars to participate online.

## Introduction

Public online spaces, such as social media, have often been promoted as promising places for teaching and learning. In the existing research on participatory cultures [1, 2], networked learning [3, 4], and various efforts to engage large and diverse audiences via networked technologies (e.g., [5, 6]), the public Web has often been envisioned as a democratizing space with the potential to foster interaction, collaboration, and civic debate [7, 8]. These positive aspects of the Web, however, are neither guaranteed nor established in all online contexts. Recent research has shown, for example, that public online spaces can reflect sociocultural biases and reinforce social stratification by yielding unequal benefits for different participants [9, 10, 11], fostering abuse and harassment [12, 13, 14], and limiting diversity of opinion and conversation by encouraging echo chambers [15, 16].

Most of the literature on the educational uses of the public Web has focused on ways to design or use online media to engender more positive learning experiences (see [17, 18]). Nevertheless, as the public Web begins to permeate every aspect of scholarly activity and students and scholars are encouraged to “go online” in order to create a digital identity and to expand the impact and reach of their scholarship [19, 20], researchers, educators, and advocates need to be cognizant of the emerging research that suggests that negativity, incivility, abuse, and harassment are prominent features of interactions on the Web. Using the public Web, and specifically social media, for educational and scholarly purposes can expose faculty and students to unwanted, rude, harassing, or generally unsociable behavior.

Thus, we were interested in how sentiment manifests itself in social spaces on the public Web. This study, therefore, was developed to gain a better understanding of the range of sentiments that scholars may face in online contexts. How likely are scholars to be exposed to negative responses when participating online? What variables might mediate their exposure to negativity? To explore attitudes expressed by audiences toward public scholarly content we build upon the work of Tsuo, Thelwall, Mongeon, and Sugimoto [21], who investigated sentiment expressed in comments toward a sample of TED talks. In particular, we asked what kind of responses were expressed toward speakers, how the gender of the speaker and the delivery format of the presentation influenced the expressed sentiment, and whether active comment moderation had any impact on the general tenor of the opinions expressed. Significantly, this is a topic of interest to a wide array of disciplines, and researchers from education, sociology, media and communication studies, human-computer interaction, and computer science may benefit from a greater understanding of the range of sentiments that individuals face in online contexts.

### Sentiment

As defined by Ortigosa, Martín, and Carro [22], sentiment is “a personal positive or negative feeling or opinion” (p. 528). Sentiment is a fact of life; everyone has feelings and opinions. Personal factors influence what people say and how they say it and in turn how people communicate and interact. However, external factors such as political, cultural, and economic climate impact sentiment [23, 24]. Given how educators might want or need to investigate topics that include or draw upon such factors as politics and economics, we must consider their impact on communication. When trending topics involve tragedy, sentiment can range from sorrow to outrage. When trending topics involve national holidays, sentiment can range from happiness to patriotism. Sentiment is important, because it can convey deeper meaning, revealing positive and negative aspects of opinions [25], personal levels of happiness or sadness [26], and even emotional health [27]. Consider the following example: An individual with a primarily conservative political ideology is more likely to possess inherently negative sentiments towards government-funded public assistance programs when they are described by, or a description of the programs includes an image of, a minority or person of color [28]. Possessing this sentiment, therefore, may negatively impact interactions in which such public assistance programs are a topic of conversation or reading. This particular situation might be more relevant to political science or sociology instructors or students, but the underlying point provides a cautionary tale for many disciplines that are impacted by sociocultural and sociopolitical phenomena.

Many people use the Internet, and specifically social media, daily to express their feelings and opinions—that is, their sentiment. Large-scale online participation coupled with the ability to capture large data sets has in turn sparked new interest in sentiment analysis or opinion mining, which Medhat, Hassan, and Korashy [29] define as “the computational study of people’s opinions, attitudes and emotions toward an entity” (p. 1093). Although early research in this area focused mostly on understanding public sentiment in relation to politics (see [30]) or products and services (see [31, 32]), researchers more recently have begun to use sentiment analysis to better understand the nuances of online communication in scholarly and educational settings [33]. It is urgent to better understand this phenomenon as scholars and students face pressures to participate online [34]. More research into sentiments expressed in online scholarly contexts will help researchers and practitioners gain a greater understanding of the actual (vis-à-vis the hoped for) sentiments that participants face online.

To investigate negative sentiment in online interactions, researchers have drawn upon a variety of communication, psychological, and educational theories. For example, researchers have explored how anonymity online increases participation while simultaneously providing an avenue for aggression and negativity [35, 36]. Using pseudonyms or impersonal screen names may allow users to interact free of fear from retribution or personal information being divulged, empowering some users to speak more candidly than they might otherwise do in person or if their identity were known. Importantly, researchers have observed a contagious contamination effect when users communicate with aggression, such as by swearing or threatening, whereby this aggressive behavior spreads to other users [37]. The festering nature of negativity in online communication sentiment appears to take on a dynamic, even cancerous-like effect. Indeed, a study by Cheng, Bernstein, Danescu-Niculescu-Mizil, and Leskovec [38] concluded that an individual's mood as well as the surrounding context of an online discussion can trigger almost anyone to engage in trolling or assume aggressive online behavior. Given the ubiquitous use of online communication, these behaviors represent a problematic challenge, particularly for educational purposes. Some learners may feel threatened or anxious when asked to engage in online conversations where masked identities exist either in whole or part, such as the case with YouTube. Alternately, potentially exposing learners to such toxicity may have negative effects: learners may succumb to the toxicity of the environment, displaying the same negative behaviors, or may face negative reactions themselves. The research on how negative sentiment manifests in online communications and how toxic sentiment impacts future communications, is only now emerging in the literature, leaving open the topic for further exploration and inquiry.

### Moderation

Comment moderation (e.g., monitoring, approving, removing, and limiting comments) has been proposed as one way to safeguard against toxic comments online and to curtail abuse. In light of increasingly negative sentiments expressed in comments on news articles and videos, including overtly and covertly racist, misogynistic, homophobic, and xenophobic statements, multiple outlets have been forced to adopt some form of moderation. Such steps have ranged from absolute, such as eliminating the ability to comment altogether, to relative, such as allowing only registered users to comment or approving all comments prior to posting [39, 40, 41].

In the case of Reddit and its many sub-communities, the outright elimination of communities that were characterized by hateful speech has also proven to be successful [39]. Some platforms have required users to register and provide personal contact information, ways to verify their identity, and how they may be contacted if need be. Such moves can raise First Amendment and privacy rights, such as when the *Buffalo News* eliminated anonymous commenting in 2010 [42]. Indeed, as noted earlier, a user’s anonymity is often associated with their ability to freely and openly express an opinion, free of retaliation or resentment [35, 43]. However, in the wake of increased efforts at online comment moderation, Ruiz et al. [44] found that moderating comments has led to two models of participation: *communities of debate* that engage in primarily respectful discussions about the topic and *homogenous communities* that express more personal feelings with little interaction among users. Whether to allow commenting and to what degree or to adopt comment moderation would thus appear to be an important issue for educators and students to consider when participating online.

### Format

The impact of particular communication formats and platforms on the sentiment expressed in online communication has just recently begun to be explored. Do different formats (e.g., video vs. text) or platforms for sharing information (e.g., YouTube vs. Twitter vs. 4chan vs. Reddit) invite or engender different sentiment or comments? The emerging research suggests that they might. In this research, we have chosen to focus on video and YouTube due to the popularity of the format and the platform with the general public. Ksiazek, Peer, and Lessard [45], found that users “are more likely to engage in user–content interaction for popular videos, but are more likely to engage in user–user interaction with less popular ones” (p. 513). In other words, if a video on YouTube is trending, users are more likely to comment on the topic in the video. However, if the video has not garnered many views or shares, comments are more likely to be directed towards other commenters or users. This particular pattern of behavior might influence whether or not an instructor chooses to use a particular video in class. The researchers further noted that public interaction metrics—such as liking, commenting, and sharing—contribute to this definition of popularity and sometimes do not reflect quality of the media content. Take for example trending videos that appear on popular cynical comedy commentary shows (e.g., *Tosh*.*0* or *Samantha Bee*). Often times, these videos are recorded and shared from mobile devices with little professional editing. These kinds of videos may have practical educational and scholarly value when placed in an appropriate context. Thus, nearly any video hosted on YouTube holds the potential for use and study.

Online communication can also be reactive or interactive, and Walther, Deandrea, Kim, and Anthony [46] found that platform differences appear to contribute to the nature of posted comments. Consider the potential difference between comments on a video uploaded directly to YouTube and a link to this video shared on Facebook. Activity on YouTube may generally trend towards direct comments about the video, including sentiment about the speaker and/or topic. These comments may or may not develop into a dialogue among users responding to one another. Comments on a Facebook post that links to this same video, however, may reveal a distinctly different trend in that comments may lead to nested replies and interactive conversations. As educators and scholars grapple with the implications of these behaviors, we return to sentiment to understand the kinds of dialogue that emerge in these environments.

### Gender

A final consideration in the context of sentiment involves the role of gender. Incidents of gendered online incivility have gained increasing research attention in recent years. Large-scale surveys in the United States by the Pew Research Center [12] and the Data and Society Research Institute [47] both found that more than 40% of surveyed Internet users had experienced some form of online abuse and that a user’s gender mediated the type of toxic comments directed at them, with women reporting more severe and sustained forms of abuse. The latter study found that women, and younger women in particular, were more likely to experience a wide variety of abuse or harassment, including purposeful embarrassment, offensive name-calling, physical threats, sexual harassment, and stalking [47]. Research indicating that women experience greater incivility and harassment across social media, online discussion forums, and multiplayer video games indicates that sentiments directed toward women are not exclusive to any one format, technology, or platform and that this study should account for the greater likelihood that women will encounter direct and indirect incivility online [48].

This study investigates participation in online spaces (e.g., posting of videos and online comments/replies) as a situated activity of facilitation, discussion, negotiation, and co-construction of knowledge (cf. [49, 50, 51]) and focuses on the sentiment expressed with respect to online videos. This framework emphasizes that digital technology is influenced by social, technological, cultural, economic, and political factors and that, accordingly, digital comments will be encouraged, restricted, and impacted by a wide array of forces (cf. [6]). In addition to the actual platform studied (i.e., YouTube), other factors that we anticipate will impact sentiments expressed in comments may include speakers’ gender, the delivery format, and comments made by other participants. This perspective aligns with the social construction of technology theory [52], which suggests that individuals’ actions shape the ways that a particular technology is used.

Furthermore, we theorize that because gender norms are policed in Western society in mainly discursive ways [53], YouTube comments and replies may be used to silence, threaten, or otherwise harass female presenters in particular [54, 55, 56]. Therefore, we expect the sentiment expressed toward female presenters to be more negative than that toward male presenters. We anticipate that the absence of visible gender markers in animations (i.e., absence of presenters in videos that consist solely of animations) will reduce this phenomenon. However, it is also likely that the presenter’s voice in animations will act as a gender cue such that gender effects remain present even after the removal of presenter’s image (cf. [57]).

## Materials and methods

### Research questions

To address the identified gaps in the literature, we posed the following research questions:

- RQ1. What is the strength of positive and negative sentiment in response to TEDx and TED-Ed Talks posted on YouTube?
- RQ2. How does the gender of the video presenter, the delivery format (presentation vs. animation), and comment threading influence the sentiment of comments and subsequent replies?
- RQ3. What would be the likely impact of moderating negative comments upon community participation?

### Context

To analyze user sentiment toward public lectures, we examined videos and comments posted in the TEDx and TED-Ed YouTube channels. YouTube is one of the most visited websites in the world [58, 21] and thus provides an authentic environment for natural experiments examining various aspects of online participation. For the purposes of this research, YouTube provides an environment to study how people communicate and specifically express sentiment toward public intellectuals such as speakers. The TEDx and TED-Ed channels are managed by TED (Technology, Entertainment, Design), an organization that hosts conferences and posts videos of speakers online for broad consumption. The content of these two channels is diverse. The TEDx channel hosts video recordings of speakers presenting to live audiences at TEDx events, while the TED-Ed channel hosts videos in animated form. The TEDx channel started in June 2009 while the TED-Ed channel was launched in March 2011. At the time of writing this article, both channels featured large numbers of YouTube subscribers (around 7.5 and 4.5 million, respectively) and video views (around 1.4 billion and 630 million, respectively). These statistics situate TED talks as successful science communication initiatives [21] and represent an accessible format from which educational spaces may be generated or extended.

The TEDx and TED-Ed channels are representative of broad public interest in educational content and public scholarship. The videos are created by experts are of high quality and are and frequently promoted for their educational value. For instance, many researchers have encouraged educators to use TED talks in their courses (e.g., [59, 60]), and the TED-Ed channel is specifically designed to be used in educational settings. The speakers who appear in TED-type talks engage in a particular form of popular science communication, and delivering a TED, TEDx, or TED-Ed talk represents a potential goal to which they might aspire. Thus, the use of TEDx and TED-Ed talks as the context for this study serves two purposes. First, designers of learning environments capitalizing on the accessibility of world renown, engaging speakers [59] may not yet fully recognize the potential pitfalls of using such a source. Second, academics and professionals who seek to communicate in these spaces may not yet fully recognize the implications of engaging in public science communication and public scholarship.

It is however significant to recognize that the particular genre represented by TEDx and TED-Ed talks is neither typical, nor does it fully capture all educational lectures on YouTube. The platform hosts a vast array of educational lectures, ranging from classroom recordings to university-created whiteboard animations to an extensive supply of educational series (e.g., *CrashCourse*, *Physics Girl*, etc). There are numerous qualitative differences between TEDx and TED-Ed talks and other types of lectures found on YouTube. Therefore, the results of this study should be understood to be bound by the context of the genre that is being investigated, and readers are cautioned to avoid drawing parallels to other types of educational video lectures.

### Data collection

We used a combination of web extraction and data mining methods to collect data. Quantitative and qualitative methods were used to analyze data. We used the YouTube API to collect data from all publicly available videos listed in the TEDx and TED-Ed channels as of February 2017. Data collected complied with YouTube’s terms of service. In total, data for 1,080 videos were collected: 570 (or 52.8%) from the TEDx channel and 510 (or 47.2%) from the for TED-Ed channel. Videos were manually coded for presenter gender, format, and delivery language. If a presenter explicitly self-identified in the talk or used a gendered pronoun in the talk description, we used those self-identifications as the gender code; otherwise, we interpreted presenter gender from names and visual appearance.

All comments for each of these videos were then collected. Many videos (33.61%) did not have any comments, suggesting that either users’ ability to comment on the video was blocked or that simply no one commented on the video. Given that the minimum number of comments among videos that received more than zero comments was 25, we determined that zero comment counts mostly likely reflected comment blocking. We considered comment counts as a binary variable of either commented or not commented and discovered a small-to-moderate Pearson correlation between format and commented, *r* = .375, *p* < .01, revealing that animations were more likely to be commented upon than talks (i.e., less likely to be blocked). However, there was no significant correlation between gender and the presence of comments. So, we concluded that comment blocking was more likely for talks than for animations but that this blocking did not vary significantly by the gender of the presenter. Thereby, we excluded videos with no comments (n = 363). We also excluded videos in languages other than English (n = 148) to simplify sentiment analysis. There was some overlap between the videos excluded, and this resulted in a final sample of 655 videos as shown in Table 1.

**Table 1:** Descriptive results of video and comment counts.

Format	Gender	Videos	Video Comments	Comment *n* Avg	Comment *n* SD
**Raw**					
**Talk**	**Female**	169	95,593	565.64	1,664.94
**Talk**	**Male**	432	261,765	605.94	2,333.35
**Animation**	**-**	479	431,360	900.54	1,649.78
		1,080	788,718	730.29	1,960.11
**Included**					
**Talk**	**Female**	75	92,932	1,239.09	2,328.55
**Talk**	**Male**	177	250,647	1,416.08	3,484.31
**Animation**	**-**	413	431,360	1,044.45	1,733.89
		655	774,939	1,165.32	2,395.08

The range of comment counts in the included videos was 25 to 31,622, M = 1,100.03, SD = 2,319.57, revealing strongly positive skew and large variation. A series of non-parametric Mann-Whitney U Tests indicated that comment counts (1) did not vary by gender but that (2) they were slightly higher for animations (Mdn = 470) than for presentations (Mdn = 390), U = 56,953.5, p = .034, and (3) were much higher for English-language videos (Mdn = 467) than for non-English videos (Mdn = 167.5), U = 8,134, p = .00. Thus, we concluded that the number of comments was not influenced by gender but that animations elicited more comments than presentations and that English-language videos elicited more comments than their non-English counterparts.

### Comment coding and sentiment analysis

We organized comments into two groups: (1) comments, representing top-level comments in response to the video, and (2) replies, representing replies to comments posted by other users. We interpreted sentiment of an item as being directed toward parent items. That is, we considered comment sentiment as directed toward the video and reply sentiment as directed toward the video and/or the parent comment.

In some of our analyses, we also incorporated a lag of up to four replies to account for replies responding to one another within the same comment thread. This was intended to account for sentiment effects over time (e.g., a negative reply engendering more negative replies). However, YouTube has not always included the reply feature, which allows users to comment in response to other comments, and previously users might respond to other comments by using an @ symbol at the beginning of the comment. Because replies did not occur before 11/7/2013, we excluded all comments from this analysis that occurred prior to this time. This reduced the total size of the dataset by only 9.23%, to 703,339, consisting of 354,539 comments and 348,800 replies.

We then generated sentiment scores for all comments and replies in the dataset, by using the open source sentiment analysis tool SentiStrength [24]. This tool has been used in prior literature, and its precision is reportedly similar to human-level accuracy (e.g., [61]). It uses a lexical approach to score the level of negativity and positivity of short social media texts. The scoring is based on identifying sentiment-related terms, linguistic rules, grammatical structures, and social media conventions (e.g., emoticons and emoji). This unsupervised approach has been shown to be better than the baseline and to have correlations very close to those used in supervised methods on YouTube comments [62]. Slightly better results for sentiment analysis have been found with supervised methods, such as linear regression, in other datasets. However, much of the accuracy of linear regression and other supervised methods relies upon domain dependence via topical terms (which is why training sets are necessary for each domain). Since individual TED Talks each likely represent their own sentiment domain (i.e., each video represents a different topic of discussion, which influences sentiment [as revealed in our first research question]), to effectively rely upon topical sentiment analysis (instead of affective terms via the lexical approach) would mean that an accurate result with a supervised method would likely require a training set for each of the separate 655 videos. Such supervision would be infeasible and would only be expected to yield minimal improvements to sentiment detection. Furthermore, prior literature argues that “the exploitation of topic is undesirable for some applications, particularly if the focus is on changes in sentiment” [24], which we seek to do here by testing how sentiment changes based upon the gender of the speaker and delivery format. To summarize, even though there might be slightly more accurate ways to determine sentiment via supervised methods that would treat each separate video as its own domain, we utilized the unsupervised, lexical approach that SentiStrength provides because (1) it allowed for topical independence across videos and (2) minor inaccuracies of the approach would theoretically be consistent across gender of presenter and delivery format (which is the focus of our analysis).

However, because sentiment is a difficult construct to measure, and even trained human coders often have difficulty coding the sentiment of artifacts with a high degree of inter-coder agreement, we sought to validate the use of SentiStrength in our dataset. In a previous study by Thelwall, Buckley, Paltoglou, Cai, and Kappas [63] that sought to validate SentiStrength, three human coders exhibited coding correlations between .56 and .68 for positive sentiment and between .64 and .66 for negative sentiment. Using the same coding instructions from the aforementioned study, four humans coded a random sample of 100 YouTube comments from our dataset for the purposes of this study. Analysis of the coded data showed similar variability of inter-coder agreement, which ranged between .59 and .8 for positive sentiment and between .66 and .78 for negative sentiment, p < .001. Based on this variability, we agreed with Thelwall et al. that an average of coders seemed to be the most reasonable method for determining sentiment strength estimates for a machine process. Pearson bivariate correlations between SentiStrength codes and average human codes exhibited moderate strength (R^2^ = .61 for positive sentiment and R^2^ = .59 for negative sentiment), which was almost identical to correlation strengths found in the aforementioned study for both positive (R^2^ = .6) and negative (R^2^ = .56) measures and outperformed other algorithmic approaches to sentiment classification explored in that study (e.g., J48, SVM, AdaBoost, Naive Bayes). Furthermore, a two-way random, absolute single measure intraclass correlation of all four human coders and SentiStrength returned a result of .55 with a 95% confidence interval from .44 to .64 (F(99,396) = 8, p < .001) on the positive measure and a result of .52 with a 95% confidence interval from .39 to .64 (F(99,396) = 8.4, p < .001) on the negative measure. By comparison, a two-way random, absolute single measure intraclass correlation of only the four human coders returned a result of .59 with a 95% confidence interval from .48 to .69 (F(99,297) = 7.92, p < .001) on the positive measure and a result of .58 with a 95% confidence interval from .43 to .7 (F(99,297) = 8.6, p < .001) on the negative measure. Taken together, we rely upon and corroborate the previous findings of Thelwall et al. that SentiStrength can provide a machine learning approach to classifying sentiment that outperforms other standard machine methods (as shown in their study) and that approaches the correlation strengths found between multiple human coders (cf. Table 2).

**Table 2:** Pearson bivariate and intraclass correlations (ICC) of human coders and SentiStrength (SS).

	Thelwall et al. (2010)		Current Study	
	Positivity	Negativity	Positivity	Negativity
**Correlation of Humans**	.56-.68	.64-.66	.59-.8	.66-.78
**Correlation of Human Avg & SS**	0.6	0.56	0.61	0.59
**ICC of Humans**	-	-	0.59	0.58
**ICC of Humans & SS**	-	-	0.55	0.52

Positivity was measured on a positive 1 to 5 scale, with 1 being neutral and 5 being extremely positive. Negativity was measured on a negative 1 to 5 scale, with -1 being neutral and -5 being extremely negative. This separation of sentiment into two separate constructs is grounded in literature in psychology [64] that proposes that sentiment is constructed by counterbalancing these two separate phenomena, which is why it is possible to experience both positive and negative emotions at the same time (i.e., mixed emotions).

Comments and replies varied by sentiment along each of the 5-point spectra. Some examples of comments and replies exhibiting varying levels of sentiment are provided in S1 Appendix. As these examples show, sentiment is a complex phenomenon, because some forms of human expression will convey nuanced messaging with both highly positive and highly negative sentiments at the same time (such as in the mixed polarity examples). For this reason, we kept negativity and positivity as separate variables for our analyses moving forward.

### Analytical strategy

To answer the question of how the sentiment of comments, gender of presenter, and form of delivery influence subsequent replies, we employed multiple regression in the framework of structural equation modeling (SEM). Multiple regression has the advantage of isolating the unique contributions of a variance of independent variables in the presence of other variables, while the SEM framework has the ability to deal with missing data and the added flexibility to deal with relationships among independent variables. The assumptions that need to be met in order for the results of multiple regression to be valid are: (a) linearity of relations between the independent and dependent variables (correct functional form), (b) independence of observations, (c) normality of the residuals, (d) equality of variance across the parameter space, (e) no extreme multicollinearity between the independent variables, and (f) no missing data in the independent or dependent variables. The assumptions of linearity, normality, and equality of variance were checked with a histogram of residuals and a residual plot. The independence assumption was expected to be violated, as there are theoretical clustering effects both at the comment and presenter level. Therefore, this was accounted for by employing multilevel modeling [65]. The SEM framework allows the independent variables to be correlated with each other, thus relaxing the multicollinearity assumption [66]. The SEM framework also deals with missing data through the full information maximum likelihood (FIML) technique, which has been shown to be more robust to missingness than listwise deletion or mean imputation [67]. All analyses were performed in the SEM program Mplus 7.4 simultaneously [68].

### Limitations and delimitations

This study faces a number of limitations and delimitations. First, the results are bound by the context of the study and may not apply to platforms other than YouTube (e.g., lectures or live video posted on Facebook or other publicly available video-hosting sites) or video types other than TED-style YouTube lectures (e.g., instructional videos, step-by-step tutorials, etc). Second, results may apply only to those presenters whose videos garner popularity or pass a certain view/comment threshold. Third, it is unclear whether TEDx and TED-Ed commenters are reflective of the larger YouTube community or of online commenters in general. The audience plays a significant factor in the results presented below, but the imprint of TED on the videos is palpable and the audience has to be understood as responding to that imprint in some way. Finally, the research focuses on computational evaluations of expressed sentiment. Sentiment itself is a complicated construct. For example, an individual can reply in an ironic—and thus positive—manner to a negative sentiment and perpetuate negativity. We have taken a number of steps to address this issue, such as for example disaggregating comments and replies and focusing our research on comments (as opposed to replies to comments) as a means to understand sentiment toward videos. The computational evaluation of sentiment via SentiStrength also faces limitations. Even though SentiStrength is not a perfect predictive tool, it is performing as well as it has performed in previous studies (wherein it outperformed other machine methods) and the evaluation results presented above show that it is reasonably accurate for how difficult a construct sentiment happens to be. Nonetheless, to gain a deeper understanding of sentiment and civility, more robust qualitative methods are necessary in future research. Though these limitations and delimitations reduce the scope of the study, we do not believe that they pose significant threats to the validity and reliability of the results presented herein. Readers should keep these in mind, however, when interpreting the results and considering how they might apply in their own contexts.

## Results

Participation in these videos tended to represent standalone commenting rather than ongoing participation by the same users. Most commenters posted one comment (Mdn = 1, M = 2.04, SD = 4.83) on one video (Mdn = 1, M = 1.38, SD = 1.43). In fact, 92.8% of users commented on only one or two videos. If a commenter left 15 comments or posted on 7 or more videos, then they were in the top 1% of participants for commenting frequency. This revealed strongly positive skew in commenting and suggested that very few participants engaged in conversations about TED talk channel videos over a period of time. We will now proceed to provide results in relation to each research question.

### RQ1. Sentiment Toward YouTube TED Talks

Across the entire dataset, 0.6% of comments and 0.67% of replies were extremely negative. Conversely, 0.28% of comments and 0.1% of replies were extremely positive (cf. Table 3). Thus, both scales revealed that most comments and replies skewed toward neutrality (rather than polarity) in sentiment, with 63.48% of comments and 58.85% of replies exhibiting no negativity, and 50.14% of comments and 57.48% of replies exhibiting no positivity. Depending upon where we set our sentiment expectations, this finding might mean very different things. A more calloused reader, for instance, might interpret this to mean that only a very small percentage of comments exhibited any form of negativity, while a more sensitive reader might interpret this to mean that one-third to one-half of comments were negative in some way. In either case, some level of negativity (e.g., disagreement) should be expected in any space where ideas are shared and explored, but extreme cases of negativity did not seem to be the norm in the dataset.

**Table 3:** Video top-level comment sentiment frequencies.

	Comments			Replies		
	**n**	354,539	50.41%	**n**	348,800	49.59%
	**Negativity**					
	**Value**	**n**	**%**	**Value**	**n**	**%**
**Most Polarized**	-5	2,140	0.60%	-5	2,339	0.67%
	-4	24,431	6.89%	-4	26,154	7.50%
	-3	34,335	9.68%	-3	42,431	12.16%
	-2	68,583	19.34%	-2	72,591	20.81%
**Most Neutral**	-1	225,050	63.48%	-1	205,285	58.85%
	**Positivity**					
	**Value**	**n**	**%**	**Value**	**n**	**%**
**Most Polarized**	5	999	0.28%	5	335	0.10%
	4	12,002	3.39%	4	5,963	1.71%
	3	67,187	18.95%	3	43,251	12.40%
	2	96,583	27.24%	2	98,748	28.31%
**Most Neutral**	1	177,768	50.14%	1	200,503	57.48%

**Fig 1:**
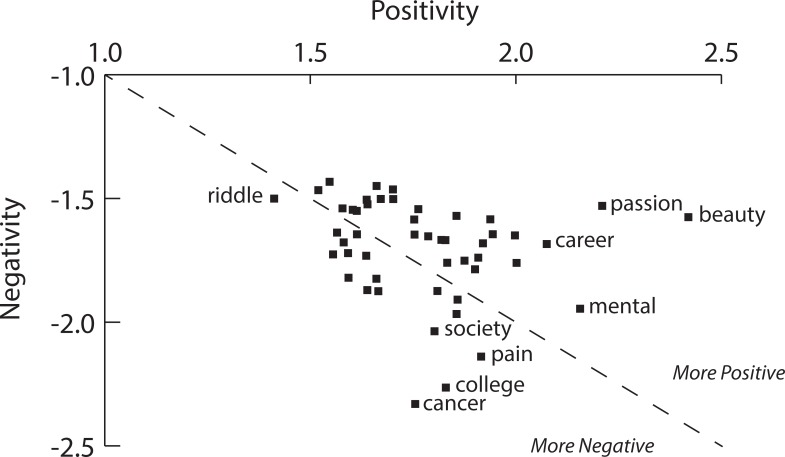
Polarity and neutrality of some common topical keywords from titles and descriptions.

As these findings emerged, we considered whether the topic of the video might mediate these results. Therefore, we conducted an exploratory analysis of sentiment toward specific topics. We extracted keywords from video titles and descriptions, ignoring stopwords such as “the” and “that,” calculated the average sentiment for videos that used common keywords, and plotted these values in Fig 1. We found that some video topics were more likely to exhibit positive sentiment in comments and replies (e.g., beauty, passion, career), while others appeared much more likely to exhibit negative sentiment (e.g., cancer, college, pain). It’s possible that positive keywords attract positive comments (and vice versa), but detailed and further analysis of these differences exceeds the scope of this study. Such investigation however would likely be fruitful for future research.

### RQ2. Influence of Gender of speaker, delivery format, and comment threading on sentiment

Sentiment analysis revealed fairly similar positivity and negativity across comments by gender and delivery format groups, with animations exhibiting the lowest absolute values and female speakers exhibiting the highest (cf. Table 4). Animations also exhibited the lowest variation in values, and female speakers exhibited the highest.

**Table 4:** Sentiment differences of comments and replies by gender of video presenter.

	Positivity		Negativity	
	M	SD	M	SD
**Animation**	1.55	0.76	-1.63	0.94
**Female Speaker**	1.94	0.94	-1.83	1.09
**Male Speaker**	1.82	0.90	-1.63	0.97

In addition to these variables, we also anticipated that the sentiment of a reply’s parent comment and the sentiment of previous replies in a comment thread might influence the sentiment of subsequent replies. For example, if one user left a very negative comment on a video, subsequent users replying to that comment might also be very negative as well. Alternatively, subsequent users might post less negative or more positive comments in response to the original negativity. To examine this hypothesis, we conducted analyses that included the sentiment of each reply’s parent comment and the sentiment of up to four previous replies as lagged variables (e.g., N-1 refers to the reply preceding the studied reply with a lag of 1). Table 5 shows the descriptive statistics of the variables used in the multilevel regression model divided up by level. The reply-level predictors show the statistics for the sentiment level of the previous replies in the thread (up to 5) as well as the cardinality (the number in the thread of the reply). The sample size of the sentiment level of the previous replies decreased as the number of replies increased. This reflects the fact that some of the threads were small and had few replies. This missingness was handled by the FIML technique. The sample size at the different levels reflects the unique number of parent comments and presentation types.

**Table 5:** Descriptive statistics of variables in multilevel regression model.

Variable	*n*	Mean	Variance	Min	Max
**Outcomes**					
**Positive Sentiment (PS) of reply**	348,800	1.69	0.61	1.00	5.00
**Negative Sentiment (NS) of reply**	348,800	-1.70	0.98	-5.00	-1.00
**Reply Level Predictors**					
**PS of previous N-1 reply**	290,501	1.58	0.60	1.00	5.00
**PS of previous N-2 reply**	253,731	1.58	0.60	1.00	5.00
**PS of previous N-3 reply**	226,608	1.57	0.60	1.00	5.00
**PS of previous N-4 reply**	205,651	1.57	0.60	1.00	5.00
**NS of previous N-1 reply**	290,501	-1.72	1.00	-5.00	-1.00
**NS of previous N-2 reply**	253,731	-1.73	1.01	-5.00	-1.00
**NS of previous N-3 reply**	226,608	-1.73	1.02	-5.00	-1.00
**NS of previous N-4 reply**	205,651	-1.73	1.02	-5.00	-1.00
**Cardinality of Reply**	348,800	20.19	1,684.15	1.00	500.00
**Comment Level Predictors**					
**Number of replies**	51,807	5.97	199.13	1.00	497.00
**Parent Comment PS**	58,107	1.70	0.73	1.00	5.00
**Parent Comment NS**	58,107	-1.84	1.12	-5.00	-1.00
**Presenter Level Predictors**					
**Male Presenter Dummy Variable**	659	0.26	0.19	0.00	1.00
**Animation Presentation Dummy Variable**	659	0.62	0.24	0.00	1.00

The assumptions of linearity, normality, and equality of variance were checked by visual inspection of the histogram of the residuals and the residual plot. This data violated the assumption of normality because of the discrete nature of the data, but as the sample size is large, the central limit theorem is invoked and MPLUS estimates its parameters with the Huber-White correction for non-normality [69]. Therefore, this violation need not be of concern. The assumption of linearity and equality of variance were not violated according to the plots.

Table 5 shows the intraclass correlations (ICCs) and the design effects (DEFFs) of the clustering effects of parent comment and presenter. Muthén and Satorra [70] demonstrated that a DEFF, which is a function of sample size and the ICCs, is a measure of the effect of clustering on parameter estimates. If a DEFF is lower than two, then one can ignore that level of clustering. Table 6 shows that the DEFFs for positive and negative sentiment were lower than two at the parent comment level. Nevertheless, this model was still included in the model to more accurately reflect the reality of the situation. The DEFFs for the presenter level were quite high as the average sample size per presenter was also high, meaning that this level needed to be included in the analysis.

**Table 6:** Design effects of positive sentiment and negative sentiment of replies at the level of parent comment and presenter.

	Intraclass Correlation	Average Cluster Size	Design Effect
**Parent Comment Level**			
**Positive Sentiment of replies**	0.06	6.28	1.33
**Negative Sentiment of replies**	0.08	6.28	1.40
**Presenter Level**			
**Positive Sentiment of replies**	0.04	603.01	22.67
**Negative Sentiment of replies**	0.06	603.01	35.31

Table 7 shows the multilevel results of the covariates on the positive sentiment and negative sentiment of replies. These results were run simultaneously in an SEM framework allowing positive sentiment and negative sentiment of a reply to covary. The results showed very little predictive power of the covariates at the reply level (R^2^ = 0.01 for positive sentiment, R^2^ = 0.01 for negative sentiment), and the standardized betas for the previous replies’ sentiments were very low. This indicated that the sentiment of previous replies and the cardinality of the reply did not predict the positive or negative sentiment of the subsequent reply.

**Table 7:** Multilevel regression results of covariates on positive and negative sentiment of replies.

	Outcome					
Predictor	Positive Sentiment (higher is more polar)			Negative Sentiment (higher is more neutral)		
	*B*	SE	*β*	*B*	SE	*β*
**Reply Level Predictors**						
**PS of previous N-1 reply**	0.05**	0.00	0.06	—	—	—
**PS of previous N-2 reply**	0.05**	0.00	0.05	—	—	—
**PS of previous N-3 reply**	0.02**	0.00	0.02	—	—	—
**PS of previous N-4 reply**	0.02**	0.00	0.02	—	—	—
**NS of previous N-1 reply**	—	—	—	0.08**	0.00	0.08
**NS of previous N-2 reply**	—	—	—	0.06**	0.00	0.06
**NS of previous N-3 reply**	—	—	—	0.02**	0.00	0.02
**NS of previous N-4 reply**	—	—	—	0.02**	0.00	0.03
**Cardinality of Reply**	0.00**	0.00	0.02	-0.00**	0.00	-0.02
**R^2**	0.01**	0.00	NA	0.00**	0.00	NA
**Comment Level Predictors**						
**Number of replies**	0.00**	0.00	-0.03	0.00	0.00	-0.01
**Parent Comment PS**	0.07**	0.00	0.42	-0.02**	0.00	-0.10
**Parent Comment NS**	-0.03**	0.00	-0.24	0.11**	0.00	0.53
**R^2**	0.27**	0.01	NA	0.31**	0.01	NA
**Presenter Level Predictors**						
**Male Presenter Dummy Variable**	-.07**	0.02	-0.52	0.10**	0.03	0.62
**Animation Presentation Dummy Variable**	-0.21**	0.02	-1.70	0.14**	0.02	0.87
**R^2**	0.44**	0.04	NA	0.08**	0.03	NA

** Indicates significance at the *p* < .01 level.

The covariates at the parent comment level were more predictive than the reply level (R^2^ = 0.27 for positive sentiment, R^2^ = 0.31 for negative sentiment). The number of replies did not statistically predict the negative sentiment of a reply (*β* = -0.01, *p* = 0.13), and while it did statistically predict positive sentiment, the standardized beta was very small (*β* = -0.03, *p* = 0.01). Parent comment positive sentiment was positively predictive of reply positive sentiment (*β* = 0.42, *p* < 0.01), meaning that positive comments begat positivity in replies; and parent comment negative sentiment was positively predictive of reply negative sentiment (*β* = 0.53, *p* < 0.01), meaning that negative comments begat negativity in replies. Each of these effects accounted for about one-half of a standard deviation in reply sentiment variance. Conversely, parent comment negative sentiment was negatively predictive of reply positive sentiment (*β* = -0.24, *p* < 0.00), meaning that less polarity in comment negativity yielded less polarity in reply positivity. Parent comment positive sentiment was weakly negative predictive of reply negative sentiment (*β* = -0.10, *p* < 0.01), meaning that polarity in comment positivity also somewhat increased polarity in reply negativity. Taken together, these results indicated that greater polarity in the comment led to greater polarity in replies in all variable instances.

The covariates at the presenter level were predictive of reply positive sentiment (R^2^ = 0.44) but were only weakly predictive of reply negative sentiment (R^2^ = 0.08). Being a male presenter led to greater neutrality in both positivity (*β* = -0.52, *p* < 0.01) and negativity (*β* = 0.62, *p* < 0.01), meaning that female presenters experienced greater polarity in replies, accounting for more than one-half of a standard deviation. Additionally, the animation format neutralized both the negativity (*β* = 0.87, *p* < 0.01) and positivity of replies (*β* = -1.70, *p* < 0.01) at a very high rate, meaning that the presenter format faced much more positivity and negativity in replies. In summary, these results indicated that presenter gender and video format influenced the sentiment of *all* replies and not just the initial comments that were directed toward the video.

### RQ3. Projected effects of moderation

Though we have no way of knowing how much moderation has already occurred in the dataset, we wanted to test the possible effect of further moderating replies and comments. Therefore, to test the effect that increased comment/reply moderation might have had on the dataset, we compared the number of replies with negative sentiments against others that occurred in response to a negative comment. We found that if moderators had set their offensiveness threshold (i.e., that which they deemed to be too negative for the community) to -5 and deleted comments that met this criteria, it would have prevented 4.36% of offensive replies and would have inadvertently also prevented 1.46% of all other replies (cf. Table 8). Though this percentage may seem low, it represents preventing non-offensive to offensive replies at a rate of 50-to-1, which means that such moderation would have impacted non-offensive replies much more than offensive replies. In contrast, if the offensiveness threshold was set to the very strict level of -2, this would have prevented 62.48% of offensive replies along with 46.63% of non-offensive replies. This represents a much higher occurrence of overall censorship (53.15% of all replies), but it also would reduce the non-offensive to offensive rate at which replies were prevented to almost 1-to-1. Herein the difficulty of comment moderation is made apparent in that the level of moderation must be weighed by its impact on both negative behaviors (offensive replies) as well as positive behaviors (non-offensive replies). Notably, however, even by employing the strictest offensiveness threshold available (-2), we were only able to prevent 68.75% of the most offensive replies (-5) and 62.48% of all offensive replies (-2 to -5). For this reason and the fact that replies overall were only slightly more negative than were comments, it seems that comment moderation based on sentiment would not be a reasonable solution to preventing offensive replies in a space like this.

**Table 8:** Projected effects of comment moderation on preventing offensive replies by threshold (-5 to -2).

Sentiment of Prevented Replies	n	%	Parent Comment Offensiveness Threshold			
			-5	-4	-3	-2
**-5 (Extremely Negative)**	2,339	0.67%	4.36%	30.74%	48.14%	68.75%
**-4**	26,154	7.50%	3.44%	32.79%	51.05%	70.92%
**-3**	42,431	12.16%	2.23%	18.68%	40.24%	63.19%
**-2**	72,591	20.81%	1.51%	14.15%	30.64%	58.83%
**-1 (Not Negative)**	205,285	58.85%	1.07%	11.27%	24.68%	46.63%
**Total Replies Prevented**			1.50%	14.51%	29.95%	53.15%
**Offensive Replies Prevented**			4.36%	32.62%	44.49%	62.48%
**Non-Offensive Replies Prevented**			1.48%	12.90%	26.24%	46.63%
**Non-Offensive to Offensive Rate**			50.33 to 1	4.45 to 1	2.31 to 1	1.07 to 1

## Discussion & conclusions

In this study, we examined the strength of positive and negative sentiment expressed in response to TEDx and TED-Ed talks posted on YouTube, the effect of several variables on comment and reply sentiment, and the projected effects that sentiment-based moderation would have had on posted content. We found that most comments and replies were neutral in nature (as opposed to positive or negative polarity) and some topics were more likely than others to elicit positive/negative sentiment. Videos of male presenters showed greater neutrality, while videos of female presenters saw significantly greater positive and negative polarity in replies. Further, animations neutralized both the negativity and positivity of replies at a very high rate. Gender and video format influenced the sentiment of replies and not just the initial comments that were directed toward the video. Finally, we found that using sentiment as a way to moderate offensive content would have a significant effect on non-offensive content. These findings have significant implications.

Results suggest that negativity in comments and replies is common. Though some past scholarship suggests that scholars may potentially face negative experiences online [71], the bulk of the literature encourages scholars to participate online and suggests positive outcomes for scholars who choose to do so [72]. Our research, however, demonstrates how common and widespread negativity may be in some online contexts. Though the results presented here are bounded by the context of the study (i.e., popular TED-style talks posted on YouTube), they nonetheless provide a benchmark against which to compare future results in other settings used for scholarly purposes (e.g., on Twitter, Facebook, personal blogs). Furthermore, the results suggest a modest effect with regards to negativity engendering negativity and positivity engendering positivity. This finding has significant implications for future design and research. Design-wise, one possible approach to reducing negativity and harassment online might be to implement early-warning algorithms that track early signs of negativity in comments to predict negativity of greater magnitude. Responses to such a warning by moderators may vary, but one potential response might be replies that encourage positivity in order to counteract negative trends. From a research perspective, it would be important to understand not just the reasons why negativity/positivity begets more negativity/positivity, but also the converse: why do some people “break the mold” and respond positively to a negative thread? What characterizes these individuals? How can social media platforms empower more individuals to respond positively to a thread that is characterized by negativity?

Results also suggest that responses to videos featuring female speakers will likely exhibit greater polarization and less neutrality than videos of male speakers. Our finding that gender mediates reactions to online participation calls into question the push to encourage all researchers to be active on social media and suggests that individuals who encourage and prepare faculty and students to participate online (e.g., faculty, faculty developers, social media trainers) should recognize that male and female faculty will have different experiences online. We encourage these individuals to help students and faculty recognize that their online participation may have differential effects based on their gender. Similarly, we urge caution in using social media comments in faculty evaluations. Further research into this finding is necessary. What other variables influence polarization? In what ways, for example, does the topic examined by the presenter influence polarization? For which topics might we see greater polarization? Is a female speaker likely to face greater polarization than a male speaker when discussing certain topics (e.g., religion or feminism)? It is also significant to note that more in-depth qualitative analysis of the results presented here may shed more light into the sentiment expressed toward YouTube videos. For instance, while we might be tempted to view increased positivity in a desirable light, it is possible that a closer inspection might reveal that increased positivity may reflect a form of harassment (e.g., in the form of cat-calling or similar behaviors).

One strategy that scholars who participate online and want a more neutral experience could employ may be to utilize an alternative video format. Results suggest that videos featuring animations as opposed to male/female speakers exhibited more neutral comments, with 0.8 S.D. less negativity and 1.7 S.D. less positivity. Therefore, in practical terms, if speakers anticipate a strong negative reaction to their work, one way to lessen such anticipated reactions might be to present their findings online in animated form. Such practice, however, puts the onus on the presenter as opposed to the commenters who post offensive content. Indeed, to reduce the incidence of negativity online, it would seem prudent to direct comments regarding steps to take to civility and respectful dialogue toward everyone who participates online—and especially those who contribute offensive content—and not simply advise content creators to change the ways they behave, speak, or present their content. Such conversations are important to have in contexts in which social media practices are explored and taught.

Finally, these results reveal that moderating comment threads has a complicated effect on participation and that such moderation does not necessarily prevent future negativity. Overly strict moderation is likely to lead to curtailing non-offensive content and to reduce positivity. Thus, algorithms and technologies developed to automate moderation and to combat offensive content should extend beyond sentiment analysis. We do wonder, however, whether efforts to moderate and limit negativity reflect a larger trend of recognizing the differential impact that negative outliers in these huge datasets of online participation have on content creators. Though extremely negative commenters represent a minority of participants identified in the dataset collected here, if their effect on content creators is significant, they may end up controlling what happens online. Regardless, some comments might have more impact than others and comments might impact some users more than others. Certain kinds of negative comments might be more toxic or hurtful than others, and some individuals might feel the effects of those comments more than others (e.g., comments that remind the content creator of previous trauma, such as sexual violence). Therefore, the case can be made here that toxic and hurtful comments should be removed for their impact, rather than just their level of negativity. The implications for practice are far reaching, but a potential first step might be the recognition that there is a need for greater investments in tools and approaches to reduce the incidence, and thereby impact, of online toxicity.

This study opens a number of significant future research directions for researchers vested in understanding and improving online interactions. Looking beyond sentiment, how do offensive, threatening, and profane comments impact participation, future replies, and presenters? Are there observable differences in how language is employed to describe women vs. men in comments to online videos? Do we see similar outcomes in other types of lectures or in other video-sharing platforms? What topics are most likely to generate polarity in online discussions? Education researchers need to develop a greater understanding of social media use in the life of scholars. How should scholars and educators prepare doctoral students, early-career researchers, and academics to navigate this space effectively and face negative sentiment online? What strategies can scholars use to curtail online negativity directed at them? If it is not possible or likely to reduce online negativity directed at them, should they respond to it? If so, what are the most effective ways to respond to online negativity? What are the ethical responsibilities of trainers and social media proponents when emerging research suggests that some research areas are rife with tensions and that people of certain backgrounds (e.g., women) are more likely to receive unsavory responses online? This research provides a starting point for delving deeply into the significant effects discovered here, and the questions posed provide fertile ground for future research.

## Supporting information

S1 Appendix Example comments and replies at varying sentiment levels. (DOCX)
