# Supplementary material for: Public comment sentiment on educational videos: Understanding the effects of presenter gender, video format, threading, and moderation on YouTube TED talk comments
Source: PLoS One. 2018 Jun 1;13(6):e0197331. doi: 10.1371/journal.pone.0197331 (PMC5983440; doi:10.1371/journal.pone.0197331)
Supplement: S1 Appendix — (DOCX) [file pone.0197331.s001.docx]

**S1 Appendix. Example comments and replies at varying sentiment levels**

| **Text** | **Negativity** | **Positivity** | **Sentiment Interpretation** |
| --- | --- | --- | --- |
| We actually wrote a paragraph from the Narcissis story. It was... PAINFUL!!!! | -5 | 1 | Very Negative |
| plus she's religious af and eveytime I tell her to go see a therapist she says that God is the only one she'll talk to that why I fucking hate religions | -5 | 1 | Very Negative |
| I have motion sickness. So, long distance journey can be unpleasant. ... | -3 | 1 | Negative |
| So where is your scientific proof she is just out to sell books? ... If she is right then 99% of studies are flawed. That pesky placebo is such a problem, isn't it? ... | -3 | 1 | Negative |
| It can be pretty annoying because the sperm cell and easily fertilize the egg all of the sudden. ... Fortunately, there are less than a hundred fumale in the world and they are only found in very few parts of South Africa. Then it's easy for their stomach to explode. ... | -3 | 3 | Mixed Polarity |
| You must be joking. ... Besides that though constructed languages are a breed all their own and they, while less of a concern than natural languages in most cases, should not be ignored. | -3 | 3 | Mixed Polarity |
| God I'm from germany and I fucking hate this techno trash... but this guy is absolutely amazing :D insane beatboxer and really funny comedian at the same time c: | -5 | 5 | Extreme Mixed Polarity |
| YET, he fucking loved every inch of his life and had no regrets, he inspired people and died doing what he loves. Dumb cunt........losers hating on him or criticizing him doesn't matter as his life was perfect and fulfilled, he found life and his soul was intact. So, go get a life bitch... | -5 | 5 | Extreme Mixed Polarity |
| This made me ask myself: Where am I going with my life? | -1 | 1 | Neutral |
| How did you know the narrator's name? Does it say somewhere? | -1 | 1 | Neutral |
| Mann I love these riddles | -1 | 3 | Positive |
| Same lmao xD | -1 | 3 | Positive |
| i really loved the closin analogy she's recited. loved her voice and smile it's influential | -1 | 5 | Very Positive |
| it's not sad :) He knows and share with us what happiness love and hope are... As so many have no idea about | -1 | 5 | Very Positive |
